# Primary Diffuse Large B-Cell Lymphoma Localized to the Lacrimal Sac: A Case Presentation and Review of the Literature

**DOI:** 10.1155/2016/5612749

**Published:** 2016-09-08

**Authors:** Kevin Zarrabi, Ved Desai, Brandom Yim, Theodore G. Gabig

**Affiliations:** Department of Medicine, Stony Brook University School of Medicine, Stony Brook, NY, USA

## Abstract

We report a rare case of diffuse large B-cell lymphoma (DLBCL) of the lacrimal sac in a 50-year-old male. The incidence of primary ocular lymphoma is low and it is considered a rare disease. Moreover, reports of ocular DLBCL are uncommon and the disease remains poorly characterized. Our patient presented for management of osteomyelitis and was incidentally found to have a painless swelling and cyst around his right eye. A PET/CT scan revealed hypermetabolic activity within the lacrimal sac and a subsequent excisional biopsy of the mass yielded histopathology consistent with DLBCL. Consequently, the patient underwent treatment with R-CHOP therapy. The patient responded well to chemotherapy with a substantial shrinkage in tumor burden and the disease remained localized. Herein, we present a rare case of primary ocular lymphoma, highlight the importance of early diagnosis, and review current treatment modalities.

## 1. Introduction

Primary malignancy of the eye is infrequent [1]. Primary lymphoma of the eye is a rarer event occurring at a rate of 0.3% per 100,000 persons in the immunocompetent population [2], with reports of various lymphoproliferative processes including extranodal marginal zone lymphoma, follicular lymphoma, DLBCL, and mantle cell lymphoma [3].

We present a case of a DLBCL originating within the right orbit, specifically within the lacrimal gland, which is a rare and atypical presentation [4]. DLBCL is an uncommon form of non-Hodgkin's lymphoma (NHL) and seldom occurs within the orbit [3] with most cases of ocular lymphoma being MALT lymphoma [5]. Our case highlights a typical presentation of this exceptionally rare disease. Moreover, studies on orbital DLBCL are limited by the lack of reported cases and the infrequency of disease prevalence. We provide a case of primary intraocular DLBCL, characterize the disease, and describe response to treatment.

## 2. Case Report

Our patient is a 50-year-old homeless male with a past medical history of uncontrolled diabetes mellitus, bipolar disorder, chronic kidney disease, left nephrectomy secondary to trauma as a child, ileal conduit placement with external bladder, and chronic pancreatitis secondary to hypertriglyceridemia who presented for management of a surgical wound leakage following a toe amputation. He was incidentally found to have a painless swelling and small cyst around his right eye that as per patient “had been growing pretty quickly.” Patient denied epiphora and diplopia but endorsed decreased visual acuity and a “sense of fullness” behind his right eye. He denied B-symptoms.

Physical exam was significant for a seven-millimeter proptosis of the right eye compared to the left, fullness around the orbit, and decreased adduction. OD examination revealed diplopia in far upgaze, downgaze, and left gaze.

**Figure 1:**
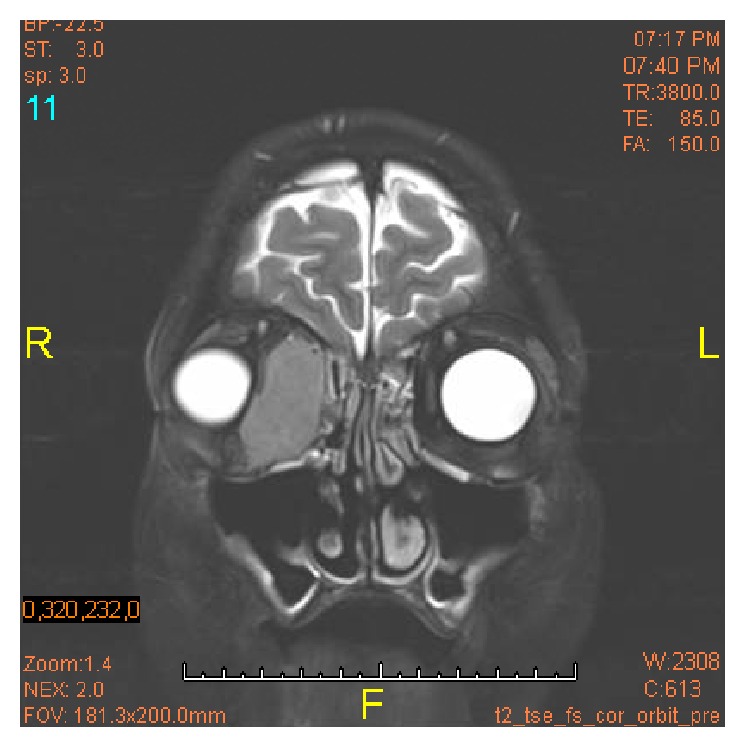
Orbits MRI. Right orbit: there is a 4.1 × 1.9 × 3.5 cm mass in the medial aspect of the right orbit. The mass displaces the medial rectus laterally and the globe anteriorly and laterally. The optic chiasm is normal in appearance. There is no suprasellar or parasellar mass. The pituitary gland is within normal limits in size.

MRI of the orbits identified a nonspecific appearing mass medial to the right orbit (Figure 1). PET/CT scan revealed primary lacrimal sac hypermetabolic activity. A right inner eye excisional biopsy was performed which yielded large B-cell lymphoma, and immunohistochemical staining characterized the cells as CD20+, BCL-6+, and CD43 (weak), Ki-76 of 80–90%. Staining was negative for CD3, CD5, CD10, CD23, BCL-2, and cyclin D-1. Bone marrow biopsy revealed no lymphoproliferative process and normal cytogenetics with no clonal B-cells or blast cells. Lumbar puncture was negative for malignant cells effectively ruling out primary leptomeningeal or leptoreticular process spreading to the orbital lacrimal sac. CT scan of the chest, abdomen, and pelvis revealed no evidence of systemic disease.

**Figure 2:**
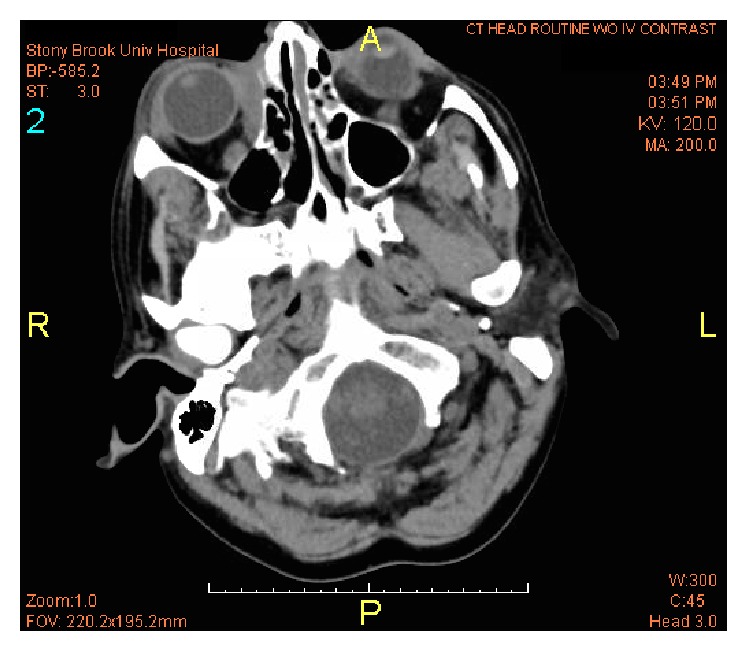
Head CT without contrast. After two cycles of R-CHOP therapy, interval CT scan revealed a significant decrease in size of right intraorbital mass lesion medially with small residual mostly in the medial extraconal space.

The cancer was staged, and the treatment plan consisted of four cycles of R-CHOP followed by radiation therapy. The patient was started on prophylactic acyclovir and underwent two cycles of R-CHOP and showed marked improvement in the size of lymphoma assessed by interval CT scans (Figure 2). The patient was discharged with outpatient completion of treatment course but was lost to follow-up.

## 3. Discussion

We report a patient with ocular NHL, which is reported to comprise less than 1% of all NHL cases [4]. The patient's biopsy revealed DLBCL, and although systemic DLBCL represents the most common lymphoma, ocular DLBCL is a rare entity [6, 7]. The patient's primary DLBCL is localized to the lacrimal sac as made evident by the PET/CT scan. Greater than 90% of nasolacrimal malignancy is epithelial in nature, and the vast majority of nasolacrimal lymphoma is secondary to metastatic disease [8]. Our patient's disease burden is due to a nonepithelial primary malignancy. To this end, the management of such a rare disease is not well characterized [9] and requires further attention.

Lymphoma of the eye is so rare that it is often diagnosed late [1]. This diagnostic delay is attributed to the nonspecific nature of the clinical signs and symptoms of the disease [4] and the first sign of disease may be destructive mass effect to ocular structures [10]. Furthermore, the painless nature of disease progression delays patients in presenting for care. This is a common obstacle to early diagnosis, which is a goal for ocular lymphoma as early diagnosis directly correlates to visual and vital prognosis [1]. DLBCL is typically high-grade and runs an aggressive course but is potentially curable [5]. The degree of p53 expression and Ki-67 antigen expression correlate significantly with clinical outcome [11]. The treatment approach to a lacrimal sac lymphoma is similar to that of the majority of NHLs. An anthracycline-based regimen, such as CHOP, is commonly employed in addition to radiotherapy. Studies have proven that anti-CD20 monoclonal antibodies are efficacious in the treatment of ocular lymphoma, and the addition of such agents has become standard of care [12]. Survival is dependent on the disease remaining localized. A recent cohort study representing the largest series of patients with orbital DLBCL in the literature found that localized disease provided an exceptionally high 5-year survival of 90.9% [7]. Advanced stage disease, however, does not provide such an optimistic survival rate. The 5-year survival from this same cohort for systemic disease was 23.5%. Although current surgical guidelines recommend surgical removal of lacrimal sac tumors, resection of orbital cell lymphoma is not recommended given the high cure rate and risk of damage to the eye [9].

Considering nearly all the recent retrospective cohort studies in the literature, a common limitation has been small sample size and weak follow-up. This is attributed to the rare nature of ocular lymphoma and even rarer nature of ocular DLBCL. Venkitaraman and George report the largest sample size consisting of data from only 37 patients collected over 10 years through a multicenter trial over four continents [9]. This demonstrates the difficulty of studying and characterizing such a rare disease. To date, multiple published studies have shown conflicting data regarding the gender predominance (male or female), average age of onset (reports range from 50 to 75 years), and 5-year survival rate (reports range from <30% to 75%) [6, 8, 13, 14]. All these aforementioned studies were limited by sample size due to low disease prevalence.

## 4. Conclusion

We report a rare case of a 50-year-old male with ocular DLBCL of the lacrimal sac diagnosed secondary to ocular proptosis and visual defect that was sensitive to R-CHOP chemotherapy and dramatically decreased in size after two treatment cycles. This case highlights a rare disease presenting in an extremely rare anatomical location. Our approach to the patient employed current diagnostic and treatment modalities.
